# Evaluation of the effectiveness of crocin isolated from saffron in treatment of burning mouth syndrome: A randomized controlled trial

**DOI:** 10.22038/AJP.2019.12764

**Published:** 2019

**Authors:** Atessa Pakfetrat, Mehdi Talebi, Zohreh Dalirsani, Ahmad Mohajeri, Roya Zamani, Ala Ghazi

**Affiliations:** 1 *Oral and Maxillofacial Diseases Research Center, Mashhad University of Medical Sciences, Mashhad, Iran*; 2 *Family Medicine Department, Faculty of Psychiatry, Mashhad University of Medical Sciences, Mashhad, Iran*; 3 *Targeted Drug Delivery Research Center, Pharmaceutical Technology Institute, Mashhad University of Medical Sciences, Mashhad, Iran*; 4 *Department of Oral Health, Gonabad University of Medical Sciences, Gonabad, Iran*

**Keywords:** Crocin, Burning mouth syndrome (BMS), Depression, Anxiety, Citalopram

## Abstract

**Objective:**

Burning mouth syndrome (BMS) is a debilitating disorder with few limited treatment modalities. Because of the proven association between BMS symptoms, and depression and anxiety, treatment modalities that alleviate the two latter etiologic factors can be clinically effective. Thus, owing to the antidepressant and potential analgesic effects of crocin (as an active constituent of saffron), the present study was performed to compare the effect of crocin and citalopram (as control) on BMS symptoms and depression/anxiety in patients with BMS.

**Materials and Methods:**

The present double-blind randomized clinical trial was carried out on BMS patients. Patients were randomly divided into citalopram (n=21) and crocin (n=26) groups and treated for 11 weeks. BMS symptoms (based on Visual Analysis Scale (VAS)), as well as anxiety and depression (based on Hamilton questionnaire) were evaluated at baseline and during the treatment period. Mann-Whitney, Chi-Square test, Independent t-test, Friedman, and Spearman correlation were employed for statistical analysis.

**Results:**

Our findings showed a significant effect for crocin on the severity of BMS symptoms, anxiety and depression in BMS patients.

**Conclusion:**

Crocin can be considered for treatment of BMS subjects with concurrent anxiety and/or depression.

## Introduction

Burning Mouth Syndrome (BMS) is a chronic orofacial pain syndrome characterized by causalgia or burning sensation in the oral cavity without any local or systemic disease (Lamey and Lamb, 1988; Netto et al., 2011). Patients often complain of changes in sensing tastes (hypogeusia, and dysgeusia) and sense of dry mouth, despite normal saliva (Bartoshuk et al., 1999; Hershkovich and Nagler, 2004). The common area affected is the tongue, but other parts of the mouth can also be involved (Carlson et al., 2000). The prevalence rate of BMS in various studies is estimated to be 0.6-15% and even up to 40% of the general population (Bergdahl and Bergdahl, 1999; Kohorst et al., 2015; Kohorst et al., 2014). This rate was reported to be 1.3% in Iranian population (Baharvand et al., 2010). The most common age of onset for this syndrome is the fifth to seventh decades of life. It often occurs in post‐menopausal women (Hakeberg et al., 1997; Sardella et al., 2006; Woda et al., 2009). The etiology of this syndrome is still unknown, but central nervous system disorders and peripheral neuropathy are known as a common cause (Borelli et al., 2010; Forssell et al., 2002; Lauria et al., 2005). Several studies showed that psychiatric factors have a significant effect on the incidence and severity of BMS symptoms. Factors such as anxiety, depression, anger, and hostility are the most important states that contribute to BMS, which can be observed in many patients with this disorder (Carlson et al., 2000; Komiyama et al., 2013). 

Although numerous agents were proposed to reduce the symptoms of oral burning in these patients, no standard treatment has been accepted yet. These agents include tricyclic antidepressants, benzodiazepines, anticonvulsants, capsaicin, alpha-lipoic acid, and selective serotonin reuptake inhibitors, as well as approaches like cognitive behavioral therapy, transcranial magnetic stimulation, and low energy diode lasers (Bergdahl et al., 1995; de Moraes et al., 2012; Femiano, 2002; Femiano et al., 2004; Gremeau-Richard et al., 2004; Heckmann et al., 2006; Kato et al., 2010; León Espinosa et al., 2004; Minguez Serra et al., 2007; Scala et al., 2003; Silvestre et al., 2012; Umezaki et al., 2015; Zakrzewska et al., 2005). Systemic treatment in these patients is often performed using selective inhibitors of serotonin reuptake such as sertraline and paroxetine (Greenberg et al., 2008). Various studies showed the effectiveness of these drugs in reducing the symptoms of BMS, but some side effects were also reported (Cole et al., 2007; Maina et al., 2002; Yamazaki et al., 2009). Citalopram as a selective serotonin reuptake inhibitor, has fewer side effects in comparison with other drugs of this group, and its efficacy in treating BMS was shown in previous studies (Ebrahimi et al., 2009; Ferguson, 2001; Miller et al., 2004). 

Medicinal plants are considered important sources of new drugs with fewer side effects than synthetic drugs. As a herbaceous plant, *Crocus sativus* L. (saffron) is used since early times due to its refreshing and anti-depressant properties (Akhondzadeh et al., 2005; Dwyer et al., 2011; Kamalipour et al., 2010; Milajerdi et al., 2016; Modabbernia and Akhondzadeh, 2013; Moshiri et al., 2006). There are various chemical compounds in this plant that exhibit pharmacological properties. Crocin, picrocrocin, and safranal are the main ingredients of saffron (Khalatbari-Mohseni et al., 2019; Ríos et al., 1996). Among these substances, crocin has been reported to be the active constituent responsible for anti-depressant, anti-anxiety and refreshing properties of saffron. Crocins, the main saffron antioxidant, are a series of mono and diglycosyl esters of crocetin that are one of the water soluble carotenoids responsible for the deep red color of dried stigmas of saffron (Abdullaev, 2002; Giaccio, 2004; Pashirzad et al., 2019; Talaei et al., 2015).

Saffron can affect the chemical transmitters such as serotonin, norepinephrine, and dopamine. These neurotransmitters can in turn, affect depression (Talaei et al., 2015). Considering the relationship between BMS and depression, the main aim of this trial was to investigate the efficacy of crocin, and compare it with that of citalopram, in the management of BMS.

## Materials and Methods

The present study was a randomized, double-blind clinical trial. The subjects were recruited from patients who referred to the Department of Oral Medicine, School of Dentistry, Mashhad, Iran. BMS diagnosis was performed by an oral medicine specialist. The criteria for selecting participants for the study were: daily deep bilateral burning sensation in the mouth for at least 4 to 6 months, persistent or increased burning intensity throughout the day, natural oral mucosa in clinical examination without any systemic or oral cause responsible for the irritation of the mouth, minimum burning intensity of 5 based on the Visual Analysis Scale (VAS) for patients with BMS and not receiving any antidepressant treatment during the last 4 weeks (Klasser et al., 2008; Scala et al., 2003). The criteria for excluding patients from the study were: a history of a systemic disease that causes burning mouth including diabetes, severe anemia, hypothyroidism, repetitious reflux or a history of micronutrient deficiency (Spanemberg et al., 2014), severe psychological disorders such as severe depression, suicide thoughts and a history of hospitalization in psychiatric hospitals, being pregnant or suspected to be pregnant, having any susceptibility to drugs or their adverse effects, current use of monoamine oxidase inhibitors, tramadol, beta-blockers, benzodiazepines, tricyclic antidepressants or during the recent months (Sardella et al., 1999).

Since BMS prevalence is not so high in Iran and there was no similar study, we were not able to determine the sample size, and the study was conducted as a pilot study with patients who were enrolled in a year (between May to December 2018).

Based on the inclusion and exclusion criteria, 47 patients entered this trial.Protocol of this research was approved by the Ethics Committee of Mashhad University of Medical Sciences (approval number: IR.mums.sd.REC.1394.66). The methodology was described for all patients and patients who were willing to participate, signed an informed consent form. At the beginning of the trial, for each patient, demographic characteristics, history of BMS and its characteristics, such as burning period and burning area were recorded.

First, the intensity of mouth burning was determined based on the VAS. In this scale, a 10 cm line evaluates pain as follows: on the left side score 0 (no pain) and on the right side score 10 (the most possible pain); the subject was asked to mark his burning pain intensity. 

Depression and anxiety symptoms were measured using the Hamilton questionnaire. All the patients had a structured interview with a psychiatrist, and psychiatric diagnoses were done based on DSM IV criteria (Diagnostic and statistical manual of mental disorder IV-text Revision).

The patients were randomized into two groups. For one group, citalopram (Sobhan Darou, Iran) was given orally once daily with an initial dose of 10 mg that increased to 20 mg after a week. For the other group, crocin tablets 15 mg (prepared by a pharmacologist) was prescribed twice daily. Both groups received the treatments for 11 weeks. Saffron stigmas were provided from Saharkhiz Co. (Mashhad, Iran). Crocin was extracted and purified from saffron stigmas using the crystallization method described in our previous study (Hadizadeh et al., 2010). Citalopram and crocin tablets were prepared using a direct compression method by the Department of Pharmaceutics, School of Pharmacy, Mashhad University of Medical Science. The shape, size and the color of citalopram and crocin tablets and their packages were similar. Packages were coded by an individual who was not involved in the study, and the mode and duration of consumption were instructed in the package leaflet. Packages were randomly delivered to patients. It should be noted that the psychiatrist, the examiner and the patients were blinded to the type of medicine prescribed. Meanwhile, patients were told to refer to the clinic in case of any side effects, for recording. Severity of burning, depression and anxiety of patients were measured and recorded at intervals of three, seven and eleven weeks.

The mean reduction in VAS, depression and anxiety scores were calculated before and after treatment for each group. Furthermore, the recovery percentage in both groups was measured according to the following formula:

Recovery percentage = [VAS T1- VAS T4]×100VAS(T1)

where the VAS score reported by the individual at the first treatment session: VAS (T1) and the VAS score reported by the individual at the final treatment session: VAS (T4).

**Statistical analysis**

For comparison of the intervening or qualitative variables, Fisher exact test and Chi-Square test were performed. Independent t-test was used for comparison of normal qualitative variables, and if the distribution of the qualitative variables was not normal, a Mann-Whitney test was carried out. Friedman test was used to analyze the effect of treatment on dependent variables over time. Pearson or Spearman correlation coefficients were used to determine the correlation between variables. Statistical analysis was performed using SPSS software (version 20) and a p<0.05 was considered significant.

## Results

Among patients with BMS who referred to Mashhad Dental School, 47 patients who had the inclusion qualification, entered the study. No patient was excluded from the groups, and all patients continued the study until the end of the treatment. The characteristics of the participants are presented in Table 1. The statistical test did not show significant difference for any of the patients’ characteristics between the two groups (Table 1).

**Table 1:** Comparison of the demographic data of the two groups at the beginning of the study

		Citalopram group	Crocin group	p value
Age (mean±SD)		48.95±1.93	52.92±1.24	p=0.08
Sex	Male	7 (33.3 %)	8 (30.8 %)	p=0.851
	Female	14 (66.7 %)	18 (69.2 %)	
Duration of BMS (Month) (mean±SD)		6.19±2.04	6.07±2.77	p=0.444
Burning severity (mean±SD)		9.0±1.30	8.9±1.20	p=0.658
Depression (mean±SD)		29.09±6.37	28.07±5.75	p=0.568
Anxiety (mean±SD)		28.76±4.02	28.08±4.37	p=0.682

At the beginning of the trial, the mean burning severity in the citalopram group was 9.0±1.30 and in the crocin group it was 8.9±1.20. Following treatment, in the eleventh week, the mean burning severity was 1.2±1.67 for the citalopram group and 1.1±1.57 for the crocin group. In both groups, the mean burning severity at the end of the study was significantly lower as compared to the values of the beginning of the trial (p<0.001), (Table 2). 

Furthermore, there was no significant statistical difference between the two groups in terms of severity of burning, neither at the beginning of the work nor at different time-points (p>0.05) (Table 2).

The mean depression score at the beginning of the study for the group that received citalopram was 29.09±6.37 and for the crocin group was 28.07±5.75. These figures decreased to 19.4±4.65 and 19.0±3.97 at the end of 11^th^ week, for citalopram and crocin groups, respectively. In both groups, the depression score at the end of the study was significantly lower than that of the beginning of the study (p<0.001). When the two groups were compared for the depression score, no significant difference was observed between the two at all time-points (p<0.05; Table 3).

**Table 2:** Mean and standard deviation of burning mouth severity (based on VAS scale) in the two studied groups during the treatment period

	Citalopram group (mean±SD)	Crocin group (mean±SD)	p value
Beginning of the study	9.0±1.30	8.9±1.20	p=0.658
Third week	4.9±2.65	5.6±2.26	p=0.387
Seventh week	3.1±2.37	2.9±2.02	p=0.652
Eleventh week	1.2±1.67 ***	1.1±1.57 ***	p=0.981

***p<0.001, eleventh week compared to the beginning of the study.

**Table 3:** Mean and standard deviation of “depression” scores in the two studied groups during the treatment period

	Citalopram group (mean±SD)	Crocin group (mean±S.D)	P value	Citalopram group (mean±SD)
Beginning of the study	29.90±6.37	28.07±5.75	P=0.568	Beginning of the study
Third week	24.45±6.01	24.5±6.05	P=0.743	Third week
Seventh week	21.6±5.39	20.92±4.96	P=0.627	Seventh week
Eleventh week	19.4±4.65 ***	19.0±3.97 ***	P=0.755	Eleventh week

***p<0.001, eleventh week compared to the beginning of the study.

The mean anxiety score was 28.76±4.02 in the citalopram group and 28.08±4.37 in the crocin group, at the beginning of the study. At the end of week 11, these scores were reduced to 18.6±5.11 and 18.0±4.38, for citalopram and crocin groups, respectively. Thus, the level of anxiety showed a significant decrease at the end of the treatment for both groups (p<0.001). It should be noted that we observed no significant difference in the anxiety scores between the two groups (p<0.05) neither at the beginning of the study nor at all time-points (Table 4). 

**Table 4:** Mean and standard deviation of “anxiety” score in the two studied groups during the course of treatment

	Citalopram group (mean±SD)	Crocin group (mean±SD)	p value
Beginning of the study	28.76±4.02	28.08±4.37	p=0.682
Third week	24.0±4.93	24.0±6.04	p=0.743
Seventh week	20.9±4.92	20.9±4.77	p=0.902
Eleventh week	18.6±5.11 ***	18.0±4.38 ***	p=0.755

***p<0.001, eleventh week compared to the beginning of the study.

**Figure 1:**
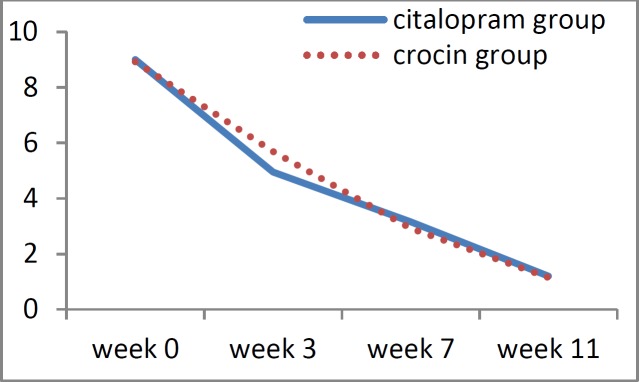
Changes in burning mouth severity based on VAS scale during the treatment, in the crocin and citalopram groups

**Figure 2:**
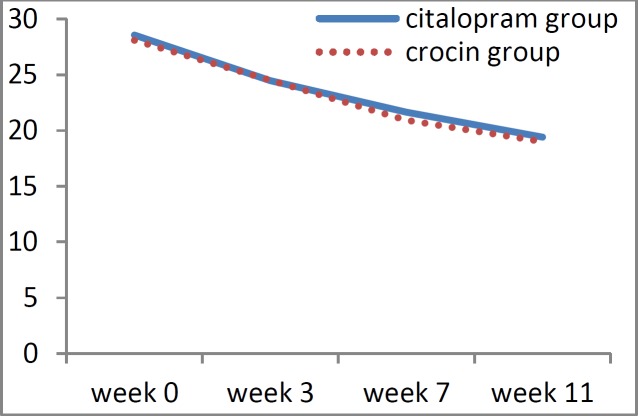
Diagram showing the trends of depression score based on Hamilton scores during the treatment, in the crocin and citalopram groups

**Figure 3:**
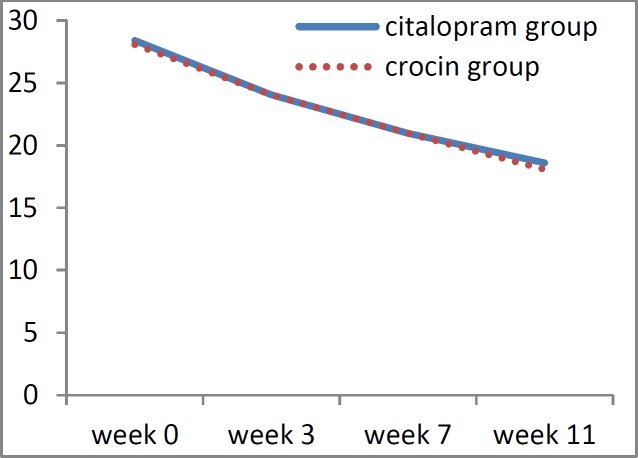
Diagram showing the trends of anxiety based on Hamilton scores during the treatment in the crocin and citalopram groups

Figures 1, 2 and 3 show the trend of changes in the severity of burning, depression, and anxiety within the two studied groups during the treatment period.

Based on the defined formula for the recovery percentage (see Methods), the average recovery percentage of mouth burning score at the end of the treatment sessions were calculated as 87.45±16.92 for the citalopram group and 87.45±16.10 for the crocin group, respectively. Moreover, when the patients' average recovery percentage of mouth burning was calculated at the third and seventh weeks and compared to the beginning of the study by using the Mann-Whitney and independent t-tests, we observed no significant difference at none of the time-points between the two groups (p>0.05).

Next, we compared the mean recovery percentage of depression score at the end of the treatment sessions with that of the beginning of the study which was proved to be 30.79±13.24 for the crocin group and 30.57±15.81 for the citalopram group. When the recovery percentages of depression were calculated at the third and seventh weeks and compared to that of the beginning of the study by applying the independent t-test, no significant difference was observed at none of the time-points between the two groups (p>0.05).

The average recovery percentage of anxiety at the end of the treatment session was 15.40±13.98 for the crocin group and 15.44±11.86 for the citalopram group. Consistently, there was no significant difference in the recovery percentage of anxiety at the third and seventh weeks between the two groups based on the Mann-Whitney test results (p<0.05). It should be noted that none of the patients in the crocin group showed signs of side effects during the trial.

## Discussion

In this study, the effect as well as side effects of crocin tablets was evaluated and compared with those of citalopram tablets for the treatment of BMS, in 47 patients with mild to severe levels of depression. The BMS cases were diagnosed by an oral medicine specialist based on standard and well-accepted criteria in the field. The effect of psychological factors including depression and anxiety on the severity of BMS is well known. Therefore, the management of symptoms in these patients requires the improvement of these underlying factors (de Souza et al., 2012; Marino et al., 2014).

Many researches have investigated the effect of saffron extract on depression and found that saffron significantly reduces the symptoms of depression (Akhondzadeh et al., 2004; Akhondzadeh et al., 2005; de Moraes et al., 2012; Hausenblas et al., 2013; Moshiri et al., 2006; Noorbala et al., 2005; Wang et al., 2010). Kashani et al. (2018) showed that saffron is effective and safe for the treatment of post-menopausal depression and hot flashes in healthy females (Kashani et al., 2018). Tabeshpour et al. (2017) also reported that treatment of minor postpartum depression with saffron over a course of eight weeks, is effective in breastfeeding mothers (Tabeshpour et al., 2017).

 Although the exact mechanism underlying saffron action is unknown, analysis of its bioactive components suggested that crocin is the main chemical responsible for the anti-depressant effect, which acts by inhibition of serotonin reuptake in synapses (Georgiadou et al., 2012; Wang et al., 2010). In animal models, several reports demonstrated that enhanced signaling of brain-derived neurotrophic factor and inhibition of selective re-uptake of serotonin by inhibitors such as fluoxetine may explain the anti-depressant mechanism of saffron (Berger et al., 2011; Ghasemi et al., 2015; Khazdair et al., 2015). The side effects of saffron have never or rarely been reported, which makes saffron a potential natural substitute in the treatment of moderate depression (Akhondzadeh et al., 2005).

In this trial, we compared the effect of crocin with citalopram for the treatment of BMS. According to recent reports, citalopram is used as the first line medicine to treat depression and anxiety (Benoliel et al., 2015). Other reasons for choosing citalopram in this comparative study were its minimal side effects and drug interactions, availability, and insurance coverage. Furthermore, different studies showed the positive effects of this medicine on the symptoms of BMS (Ebrahimi et al., 2009). Miller et al. (2002) evaluated the effect of citalopram on depression, anxiety and burning in BMS patients. In this study, citalopram (20 mg per day) was prescribed to patients for six weeks; citalopram improved depression, anxiety and burning mouth which is in accordance with our results. However, in our study, the treatment period was longer and it resulted in greater reduction of BMS symptoms (Miller and Rabe-Jablonska, 2003).

The results of this study demonstrated no significant difference in the severity of burning between the two studied groups at different time-points, and the mean burning severity decreased over the course of 11 weeks. In addition, no significant difference was observed in the severity of anxiety and depression between the two groups at all time-points. Consistently, we observed a significant decrease in levels of depression and anxiety in both groups after treatment with crocin or citalopram during the study period. Therefore, it seems that the effect of crocin on the severity of burning, depression, and anxiety in BMS patients is similar to that of citalopram. Previous studies indicated a significant relationship between depression and anxiety levels and BMS symptoms, as well as between the treatment of these two causative factors and reduced severity of BMS symptoms in patients (Botha et al., 2001; Lamey et al., 2005; Pokupec-Gruden et al., 2000; Sardella et al., 1999). Likewise, in the current study, we found that concomitant with the improvement of depression and anxiety, the severity of burning mouth decreased. This is the first study that compares the effect of crocin and citalopram tablets in the treatment of BMS patients, hence direct comparison of our findings with data reported by previous studies is not possible. Nonetheless, the results of this study are similar to those reported by a number of previous reports that evaluated crocin effects on anxiety and depression and compared them with those of anti-depressant medicines such as citalopram and fluoxetine (Akhondzadeh et al., 2010; Amin and Hosseinzadeh, 2012; Ghajar et al., 2017; Kashani et al., 2017; Mazidi et al., 2016). Ghajar et al. (2017) assessed the effect of saffron and citalopram on major depressive symptoms and anxiety disorders. Within six weeks, both treatments significantly improved the symptoms of anxiety and depression but no statistically significant difference was observed between the two treatments in terms of reducing the symptoms (Ghajar et al., 2017). In other studies, crocin effect was compared with that of fluoxetine, and the results proved a significant effect of crocin on minor and major depression (Ghajar et al., 2017; Kashani et al., 2017; Sahraian et al., 2016). The effects of serotonin and inhibitors of norepinephrine reuptake (such as citalopram) are clinically apparent one to two weeks after the initiation of treatment; therefore, to assess the effect of these compounds on various disorders, a longer treatment interval should be considered (Ghajar et al., 2017; Kashani et al., 2017). Here, an 11-week treatment was considered to study the effects of citalopram and crocin on BMS that appears to be appropriate given the satisfactory results. 

The effect of crocin on anxiety was investigated both in animal models and humans. Pitsikas et al. (2008), using an animal model, and Fukui et al. (2011) in clinical settings, evaluated the effect of saffron odor on anxiety. Both studies showed that treatment with saffron extract and crocin for more than a week leads to a significant improvement of anxiety symptoms in both animals and humans (Fukui et al., 2011; Pitsikas et al., 2008).

Our results also confirmed that long-term administration of crocin (similar to citalopram) leads to improvement of anxiety symptoms in patients with BMS

An important limitation of the current trial was the lack of long-term follow-up for the effect of crocin on the severity of BMS symptoms, depression and anxiety. For future studies, it is suggested to assess the effect of long-term administration of crocin on these symptoms with a larger sample size.

The findings of this trial showed that crocin, in addition to treating mouth burning in BMS patients, reduces the depression and anxiety levels in these patients and these effects are similar to those resulted from citalopram. According to the results of our study, crocin treatment can be suggested as a therapeutic method for BMS patients who also show signs of depression or anxiety.
